# Neonatal *Streptococcus pneumoniae* infection induces long-lasting dysbiosis of the gut microbiota in a mouse model

**DOI:** 10.3389/fmicb.2022.961684

**Published:** 2022-08-18

**Authors:** Yuanyuan Li, Ximing Xu, Ziyao Guo, Qinyuan Li, Yiying Wang, Ding Jian, Guangli Zhang, Xiaoyin Tian, Shiyi Chen, Zhengxiu Luo

**Affiliations:** ^1^Department of Respiratory Medicine of Children’s Hospital of Chongqing Medical University, National Clinical Research Center for Child Health and Disorders, Ministry of Education Key Laboratory of Child Development and Disorders, Chongqing, China; ^2^Chongqing Key Laboratory of Pediatrics, Chongqing Key Laboratory of Child Infection and Immunity, Chongqing, China; ^3^Department of Medical Record Statistics of Children’s Hospital of Chongqing Medical University, National Clinical Research Center for Child Health and Disorders, Ministry of Education Key Laboratory of Child Development and Disorders, Chongqing, China; ^4^Chongqing Higher Institution Engineering Research Center of Children’s Medical Big Data Intelligent Application, Chongqing, China

**Keywords:** long-lasting dysbiosis, mice, gut microbiota, neonatal infection, *Streptococcus pneumoniae*

## Abstract

Early life is a “critical window” for gut microbiota development, antibiotic use during this period exerts a profound effect on gut microbial dysbiosis and asthma. In clinical practice, antibiotics are usually used in patients with bacterial infections, we previously showed that neonatal *S. pneumoniae* pneumonia promoted adult-onset asthma in mice model, while it remains unclear whether neonatal *S. pneumoniae* infection have long-term effects on gut microbiota. Neonatal BALB/c mice were inoculated with 5*10^6^ CFU D39 to establish non-lethal *S. pneumoniae* pneumonia model. At 2, 3, 8 weeks of age, feces in the cecum were prepared for 16S rRNA sequencing, lungs were collected for histopathologic and lung function analysis. *S. pneumoniae*-infected neonatal mice exhibited histopathologic lesions in their lungs and increased airway hyperresponsiveness, obvious alterations in alpha and beta diversities in the entire gut microbiota, and changes of the community structure during the breastfeeding period, infancy, and adulthood. Furthermore, gut microbial composition was modified after neonatal *S. pneumoniae* infection, with a decreased relative abundance of Lactobacillus in the breastfeeding period and infancy; in adulthood, the relative abundance of Allobaculum diminished while that of Proteobacteria was augmented. Neonatal *S. pneumoniae* infection induced a long-term alteration in microbial community composition.

## Introduction

The microbiome consists of organizations of bacteria, viruses, archaea, and fungal species, majority of which reside in the gut. The acquisition of the gut microbiota begins at birth and reaches a relatively stable level at nearly 3 years of age in humans and at 8 weeks of age in mice (Laukens et al., 2016); the gut microbiota play key roles in homeostasis, metabolism, immunity, and physiologic or pathologic situations. The formation of the gut microbiota reflects several stages, and early life is hypothesized to be the “critical window” for gut microbiome development, that is, when gut microbial dysbiosis exerts the greatest influence on immune and metabolic systems (Cunningham et al., 2014; Houghteling and Walker, 2015). Microbial dysbiosis during this period is linked to various types of immune-related diseases such as asthma (van Nimwegen et al., 2011; Arrieta et al., 2015; Depner et al., 2020; Gürdeniz et al., 2022), eczema, and food allergies (Azad et al., 2015). Microbial dysbiosis in the early years of human life is associated with a variety of factors—including delivery mode, feeding pattern, and antibiotic use. Accumulating evidence reveals that early-life use of antibiotics is a risk factor for microbial dysbiosis in both human trials and in mouse models (Penders et al., 2011; Nobel et al., 2015; Stokholm et al., 2018). In neonates, the development of the gut microbiota differs between antibiotic-treated and non-treated infants (Tanaka et al., 2009; Fouhy et al., 2012), indicating a long-lasting alteration in the microbiome due to antibiotic use, and antibiotics are typically administered clinically to patients with bacterial infections. However, whether or not bacterial infection itself influences the microbiota composition needs to be further explored. Studies have indicated that influenza (Ding et al., 2019), HIV (Gosmann et al., 2017), RSV (Groves et al., 2020), and *Candida albicans* (d’Enfert et al., 2021) infection influence disease severity through modifications in the composition of the gut microbiota. Yet, whether early-life bacterial infection affects gut microbiota development is less well understood.

*Streptococcus pneumoniae (S. pneumoniae)* is the most common bacterial pathogen of community-acquired respiratory infection in childhood (Fritz et al., 2019). Our previous studies showed that even when *S. pneumoniae* was eliminated from the lung tissue 7 days post-infection, neonatal *S. pneumoniae* infection still promoted asthma development in adults (Yang et al., 2015) by alerting CD4^+^T cell subsets (Tian et al., 2020), indicating that neonatal infection with *S. pneumoniae* induced long-term effects on pulmonary immune function. However, whether neonatal *S. pneumoniae* infection promoted long-term immune alterations in the lungs by perturbing the gut microbial community remains unclear.

Against the backdrop of early-life events in the development of gut microbiota and asthma, we herein investigated the dynamic changes in gut microbiota after neonatal *S. pneumoniae* infection in a mouse model. We conducted 16S rRNA gene high-throughput sequencing in feces collected from mice 1 week post-infection (1 wpi, breastfeeding period), 2 weeks post-infection (2 wpi, infancy), and 7 weeks post-infection (7 wpi, adulthood) after nasal inoculation. Our results indicated that neonatal *S. pneumoniae* infection induced a marked perturbation in the microbiota at different developmental stages, especially in infancy. With this study, we first advanced the concept that early-life bacterial infection promoted a gradual but long-lasting influence on gut microbiota. We expect that these results will be helpful in better understanding early-life bacterial infection as related to microbiome disturbances and in providing guidance for developing potential interventions in infection-induced asthma.

## Materials and methods

### Animals

Parturient BALB/C mice and their newborn pups were purchased from Ensiweier Biotechnology Co., Ltd. (Chongqing, China). All experimental protocols were approved by the Institutional Animal Care and Research Advisory Committee of the Chongqing Medical University, and all animals were treated in accordance with the guidelines issued by the Chinese Council on Animal Care. Newborn mice were housed with their mother at 25°C under a 12-h light/12-h dark cycle with a normal diet and water.

### Study design

**FIGURE 1:**
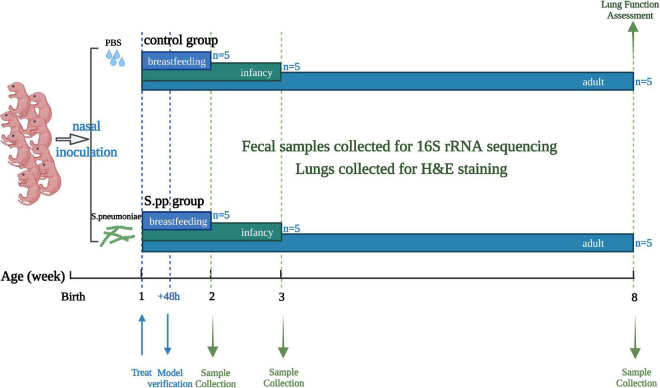
Experimental design and sample-collection time. In brief, mice were intranasally instilled with PBS (Control) or *Streptococcus pneumoniae* (S.pp) at 1 week of age, and a lung homogenate was used for model verification 48 h post-infection. Fecal and lung samples were collected at 1 wpi (breastfeeding period), 2 wpi, (infancy), and 7 wpi, (adulthood) for 16S rRNA sequencing and H&E staining, lung function were assessed in adulthood.

Totally 32 neonatal mice at 1 week of age were randomly allocated to two groups: the mock-infected control group (mean weight, 5.22 ± 0.54 g; F/M, 8/7; 10 ul of PBS) and the *S. pneumoniae* infection (S. pp) group (mean weight, 5.30 ± 0.36 g; F/M, 8/7; *S. pneumonia* = 5*10^6^ CFU in 10 ul of PBS). Forty-eight hours post-infection, two infected mice were randomly selected for a model verification test. Then, at 1 wpi (2 weeks old, breastfeeding period), 2 wpi (3 weeks old, infant), and 7 wpi (8 weeks old, adulthood), five mice were randomly selected from each group and euthanized, and the feces in their cecum and lungs were collected. The flow diagram of the experimental design is depicted in Figure 1. Our infectious model was verified with tissue homogenates cultured on blood agar (Supplementary Figure 1) (Dong et al., 2020). The fecal samples were collected for 16S rRNA sequencing, and lungs were collected for histopathologic staining.

### Establishment of a non-lethal neonatal *Streptococcus pneumoniae* infection model

The non-lethal neonatal *S. pneumonia* infection model was established according to our previous study (Yang et al., 2015; Tian et al., 2020). Briefly, the *S. pneumoniae* D39 strain was plated onto blood agar (Bio-Caring, P0901, China) and incubated for 12–14 h at 37°C in 5% CO_2_ in air. Bacteria were then transferred to Todd-Hewitt (BD, BX04921, United States) broth supplemented with 0.5% yeast extract (THY) and incubated at 37°C until the mid-logarithmic phase (OD_600_ = 0.50–0.55). Neonatal (1-week-old) BALB/C mice were inoculated intranasally with 10 μL bacteria suspension (5*10^6^ colony-forming units (CFU) of *S. pneumoniae* in PBS) without anesthetic, while the mock-infected controls received only the same volume of sterile PBS. Forty-eight hours post-infection, two infected mice were randomly selected, and their lungs were removed and homogenized; the tissue homogenates were then cultured on blood agar for 12–16 h at 37°C under 5% CO_2_ in air to determine the bacterial load.

### Sample collection, DNA extraction, and amplification

Mock-infected controls and neonatal *S. pneumoniae*-infected mice were euthanized by cervical dislocation at 2 weeks (1 wpi, breastfeeding period), 3 weeks (2 wpi, infancy), 8 weeks (7 wpi, adulthood) of age; and the mouse age brackets were classified according to a related review (Sengupta, 2013). The contents of the cecum were collected and stored at –80°C for analysis of microbial composition. Total genomic DNA was extracted from the collected fecal samples (∼0.1 g) using the OMEGA Soil DNA Kit (M5635-02) (Omega Bio-Tek, Norcross, GA, United States) following the manufacturer’s instructions and immediately stored at –20°C for further analysis. The hypervariable V3–V4 region of the bacterial 16S rRNA genes was amplified by PCR using the forward primer 338F (5′-ACTCCTACGGGAGGCAGCA-3′) and the reverse primer 806R (5′-GGACTACHVGGGTWTCTAAT-3′). Each 25-μL PCR reaction contained 5 μl of buffer (5 ×), 0.25 μl of Fast pfu DNA Polymerase (5 U/μl), 2 μl (2.5 mM) of dNTPs, 1 μl (10 uM) of each forward and reverse primer, 1 μl of DNA template, and 14.75 μl of ddH2O. The thermal cycling conditions consisted of an initial denaturation at 98°C for 5 min, followed by 25 cycles of denaturation at 98°C for 30 s, annealing at 53°C for 30 s, and primer template extension at 72°C for 45 s, with a final extension of 5 min at 72°C.

### 16S rRNA sequencing and taxonomic classification

PCR amplicons were purified and quantified using the Illlumina NovaSeq platform with the NovaSeq 6000 SP Reagent Kit (500 cycles) at Shanghai Personal Biotechnology Co., Ltd., Shanghai, China. QIIME2 was slightly modified according to the official tutorials and used for quality filtering of DNA sequences, demultiplexing, taxonomic assignment, and alpha and beta diversity calculations. Non-singleton amplicon sequence variants (ASVs) were aligned and used to construct a phylogeny with fasttree2. Taxonomy was assigned to ASVs using the classify-SciKit Learn’s sklearn naiïve Bayes taxonomy classifier in the feature-classifier plugin against the Greengenes Release 13.8 Database.

### Bioinformatics and statistical analyses

Data analyses were executed using QIIME2 and R packages (v3.2.0), and the microbiota alpha-diversity indices were calculated using the ASV table in QIIME2 and visualized as box-plots. To investigate the structural variations in microbial communities across samples, beta-diversity analysis was employed and visualized *via* principal coordinate analysis (PCoA) and non-metric multidimensional scaling (NMDS). Differentiation of microbiota structure among groups was assessed by permutational multivariate analysis of variance (PERMANOVA). The taxonomic compositions and abundances were visualized using MEGAN and GraPhlAn. Linear discriminant analysis effect size (LEfSe) was exploited to detect differentially abundant taxa across groups using the default parameters. We implemented co-occurrence network analysis with SparCC, and the pseudocount value in SparCC was set to 10-6. The cut-off value for correlation coefficients was determined to be 70 through random matrix theory-based methods as implemented in the R package RMThreshold. Based on the correlation coefficients, we constructed a co-occurrence network with nodes that represented ASVs and edges that represented correlations between the ASVs. We visualized the network using the R packages igraph and ggraph. Microbial functions were predicted by Phylogenetic Investigation of Communities by Reconstruction of Unobserved States (PICRUSt2; Douglas et al., preprint) with MetaCyc. One-way analysis of variance (ANOVA) and Kruskal–Wallis tests were used to compare the relative abundances among groups with Graphpad Prism 9.0.0.

### Histology of the lungs

After collection, the lungs were fixed in formaldehyde for 24 h and then dehydrated and paraffin embedded. The lung tissues were sectioned at 4 microns and then stained with hematoxylin and eosin stain (H&E; Sigma-Aldrich). At least three views were then selected from each mouse for analysis. Images were captured under a Nikon Eclipse E200 microscope connected to a Nikon Coolpix 995 camera (Nikon, Tokyo, Japan).

### Airway hyperresponsiveness measurement

As previously described, airway hyperresponsiveness (AHR) was measured *in vivo* by Penh using whole-body plethysmograph method. Penh is a dimensionless value represents pulmonary airflow resistance, which also reflects a function of the timing of expiration, and the ratio of peak expiratory flow to peak inspiratory flow. Briefly, each conscious adult mouse was exposed to aerosolized normal saline followed by increasing concentrations of aerosolized methacholine (Sigma-Aldrich, St. Louis, MO, United States) solution (3.125, 6.25, 12.5, and 25 mg/ml; Sigma-Aldrich, St. Louis, MO, United States) in saline for 3 min and then rested for 2 min. The average Penh for each concentration was calculated from the continuously recorded pressure and flow data for 5 min.

## Results

### Neonatal *Streptococcus pneumoniae* infection induces pulmonary histopathologic changes

**FIGURE 2:**
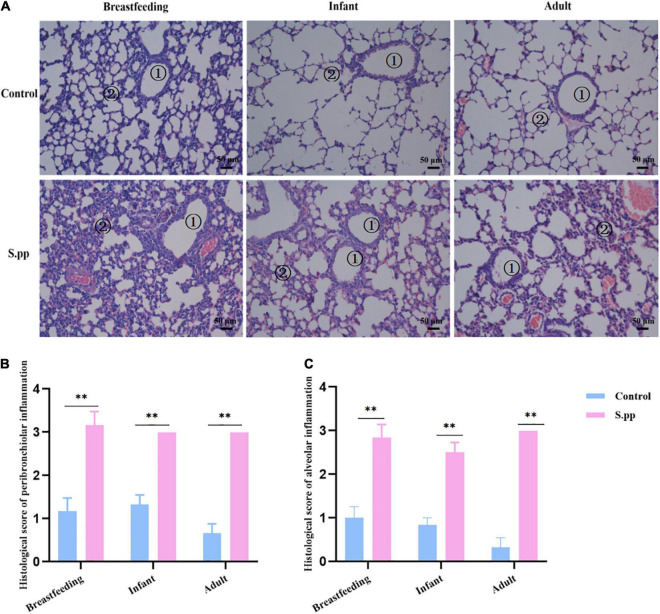
Neonatal *S. pneumoniae* infection induces inflammatory cell infiltration in lung tissues at different stages. **(A)** Representative H&E staining in lung slices from mice in the control and S. pp groups during the breastfeeding period, infancy, and adulthood (200 × magnification). The numbers in the figures indicate bronchioles ➀, alveoli ➁. **(B)** Histological scores of pulmonary peribronchiolar inflammation. **(C)** Histological scores of pulmonary alveolar inflammation. *n* = 3–6 mice/group. **P < 0.01, compared with control group.

As we previously found, *S. pneumoniae* was eliminated from the lung 7 days post-infection. Simultaneously, infected mice gained transient body and lung weight losses along with *S. pneumoniae* infection within the first-week post-infection, while restored at 14-days post-infection (Peng et al., 2019). The lungs were further collected during the breastfeeding period (1 wpi), infancy (2 wpi), and adulthood (7 wpi). As shown in Figure 2, compared with the mock-infected control group, lung sections of the neonatal *S. pneumoniae* infection group during infancy and adulthood showed histopathologic lesions that included increased inflammatory cell infiltration and thickened alveolar septa. These results indicated that neonatal *S. pneumoniae* infection induced long-lasting structural changes in the pulmonary tissue.

### Neonatal *Streptococcus pneumoniae* infection promotes long-lasting airway hyperresponsiveness

**FIGURE 3:**
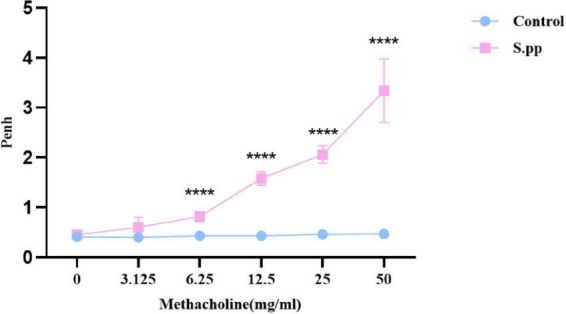
Neonatal *S. pneumoniae* infection promotes long-lasting AHR. Wholebody plethysmography was conducted in mock-infected (control) and neonatal *S. pneumoniae* infection mice (S.pp) with methacholine challenge. All data are presented as mean ± SD (*n* = 5 mice/group). ****P < 0.0001, compared with control group.

Seven weeks after *S.pneumoniae* infection, AHR was measured by the calculation of Penh. The airway responsiveness appeared no significant differences following normal saline challenge. While with the methacholine concentration increase, neonatal *S. pneumoniae* infection significantly increased AHR when compared to the controls at methacholine concentrations of 6.25,12.5, 25.00, and 50.00 mg/ml (Figure 3). These indicated that neonatal *S. pneumoniae* infection promoted long-lasting airway hyperresponsiveness.

### Neonatal *Streptococcus pneumoniae* infection alters gut microbial diversity and community structure

Based on our high-throughput sequencing, a total of 2,194,529 raw reads were generated for 30 fecal samples. After removing the low-quality sequences, 1,696,576 high-quality reads were obtained for abundance analysis, diversity analysis, and taxonomic comparisons.

#### Alpha diversity

**FIGURE 4:**
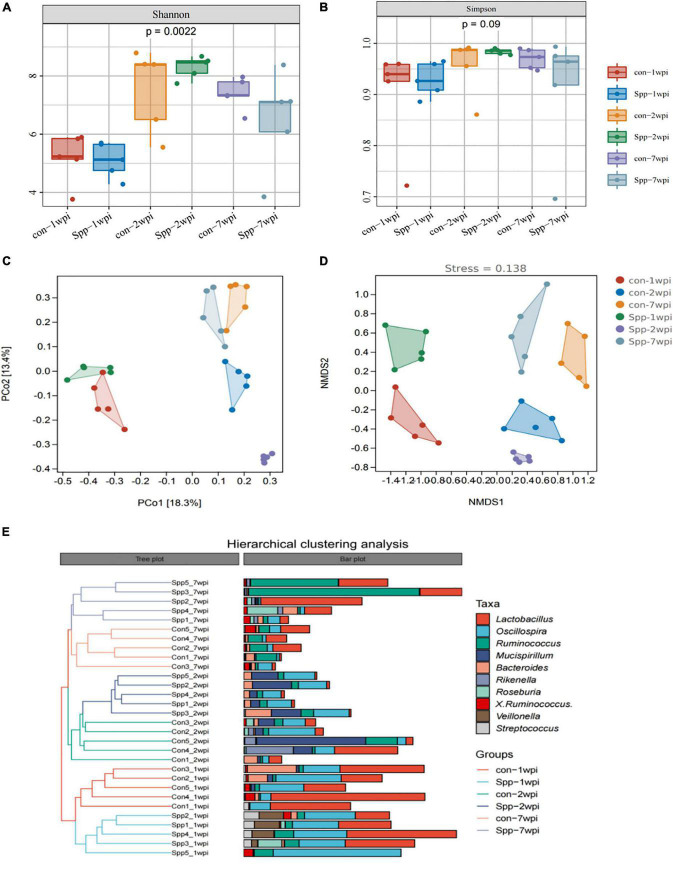
Neonatal *S. pneumoniae* infection alters gut microbial community structure. **(A)** The alpha diversity of gut microbiota from the mock-infected control- and neonatal *S. pneumoniae* infection (*S*. pp)-treated mice in the breastfeeding period (1 wpi), infancy (2 wpi), and adulthood (7 wpi) was represented by Shannon indices. **(B)** The alpha diversity of gut microbiota from the mock-infected control- and neonatal *S. pneumoniae* infection (*S*. pp)-treated mice was represented by Simpson indices at different stages. **(C)** Beta-diversity analysis of relative abundances of the entire microbiota using principal coordinate analysis (PCoA) with Bray-Curtis dissimilarity index, followed by the PERMANOVA significance test. **(D)** Beta-diversity analysis of whole-microbiota relative abundance with two-dimensional non-metric multidimensional scaling of Bray-Curtis dissimilarity distance metric (2D stress value = 0.138). **(E)** Hierarchical clustering of mock-infected control- and *S*. pp-treated mice in the breastfeeding period, infancy, and adulthood.

As evident from both Shannon and Simpson indices, the alpha diversities across different age groups exhibited an “inverse U” trend that significantly increased during infancy (2 wpi) and then slightly diminished during adulthood (7 wpi), with a significantly altered overall Shannon index (*p* = 0.0022, Figure 4A) and Simpson index (*p* = 0.09, Figure 4B). When compared with the mock-infected control mice at the same age, the reducing trends were observed in the neonatal *S. pneumoniae* infected group, further PERMANOVA test revealed that not only age but also *S. pneumoniae* infection induced significant differences in alpha diversity indices (*p* = 0.001, Supplementary Figure 2 and Supplementary Table 1).

#### Beta diversity

Principal coordinate analysis and NMDS analyses were applied based on ASV/OUT for beta diversity analysis. As indicated, the plots reflecting the samples taken during the breastfeeding period (1 wpi), infancy (2 wpi), and adulthood (7 wpi) were discretely separate, as were the plots for the neonatal *S. penumoniae* infection group relative to the mock-infected controls at the same age (Figures 4C,D). PCoA was also implemented based on the phylum, order, class, family, genus, and species, and we demonstrated that the more granular the hierarchy, the more pronounced the distinctions (Supplementary Figure 3). The plots of the samples in infancy were most markedly disparate for each hierarchy.

#### Hierarchical cluster analysis

We subsequently constructed a dendrogram using hierarchical cluster analysis with Bray-Curtis dissimilarity indices, and as shown in Figure 4E, the samples from breastfeeding mice were grouped into one major branch of the sample tree, while the subgroups of infant and adult mice co-clustered as another major branch. Moreover, for every sub-branch for age, the neonatal *S. pneumoniae* infected group and the mock-infected controls were clustered discretely. These results portrayed neonatal *S. pneumoniae* infection as exerting a significant effect on the structure of the gut microbial community.

### Neonatal *Streptococcus pneumoniae* infection affects gut microbial composition

**FIGURE 5:**
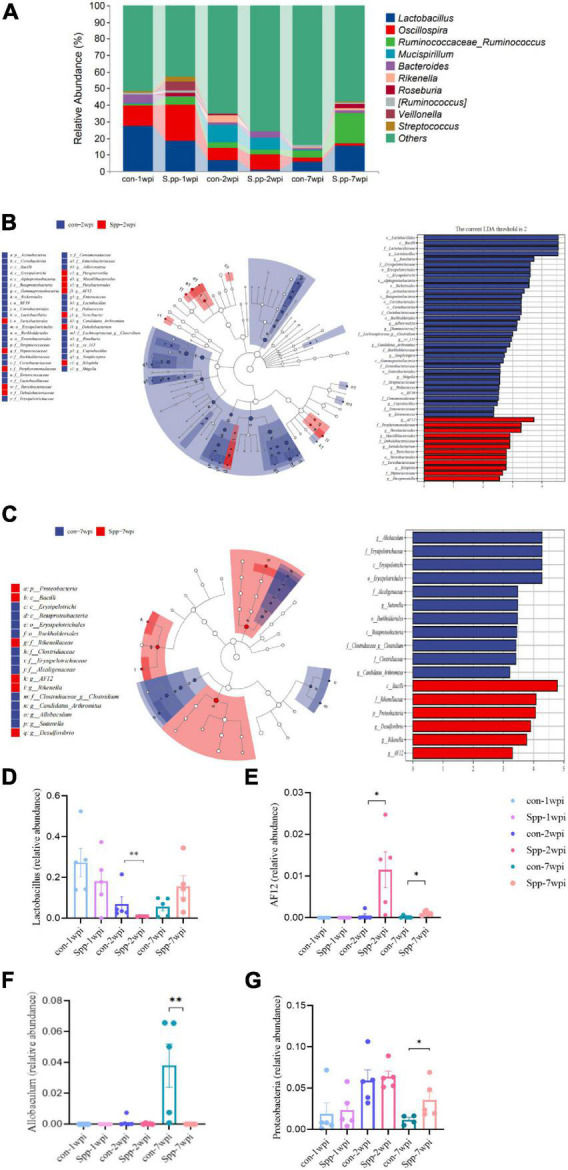
Effects of neonatal *S. pneumoniae* infection on gut microbial composition. **(A)** Relative abundances at the genus level of the mock-infected control and neonatal *S. pneumoniae* infected (*S*. pp) groups in the breastfeeding period (1 wpi), infancy (2 wpi), and adulthood (7 wpi). **(B)** LEfSe analysis based on the samples of the control and *S*. pp groups in infancy in various abundant taxa. The left panel shows the cladogram and the right panel shows the linear discriminant analysis (LDA) score (LDA > 2 shown in the Figure) for indicator taxa of the control and *S*. pp groups (*P* < 0.05). **(C)** LEfSe analysis based on the samples of the control and *S*. pp groups in adulthood in various abundant taxa. **(D)** Relative abundance of the genus *Lactobacillus* was significantly decreased in infancy (^**^P < 0.01, con-2wpi vs. Spp-2wpi); **(E)** Relative abundances of the genus *AF12* were significantly increased in infancy and adulthood (*P < 0.05, con-2wpi vs. Spp-2wpi, con-7wpi vs. Spp-7wpi; **(F)** Relative abundance of the genus *Allobaculum* was significantly reduced in adulthood (*P* < 0.01); **(G)** Relative abundance of the phylum Proteobacteria was significantly reduced in adulthood (*P* < 0.05).

As a consequence of the altered microbial-community structure, we further explored microbial composition, and our analysis of taxonomic composition revealed that the most prevalent phyla were Firmicutes (74.15%) and Bacteroidetes (18.59%) (Supplementary Figure 4), while *Lactobacillus* (12.40%) was the most prevalent genus in the microbial community. *Lactobacillus*, which is considered to be an probiotic genus, showed the highest relative abundance in the breastfeeding period, was attenuated in infancy, and then remained relatively stable until adulthood. When compared with their respective mock-infected control group, samples of neonatal *S. pneumoniae* infected groups exhibited a reduced trend for *Lactobacillus* in the breastfeeding period and a significant reduction in relative abundance in infancy, even when the brief changes in composition recovered in adulthood (Figure 5A). These modulation patterns indicated the long-lasting disruption of gut microbial community composition by neonatal *S. pneumoniae* infection and one that occurred gradually.

To explore the contributions of the differential flora on gut microbial alterations, the differences in the relative microbial abundances within the gut microbiota in samples were determined using LEfSe analysis (Figure 5B). The results revealed that the most notably enriched taxa were *Lactobacillales* bacilli, *Lactobacillaceae*, and *Lactobacillus* (all belong to Firmicutes) in the mock-infected control group, while *AF12* (belonging to Bacteroidetes) was the most enriched taxa in the neonatal *S. pneumoniae*-infected group in infancy. In adulthood, the most obviously predominant taxa in the control group were *Allobaculum*, *Erysipelotrichales*, *Erysipelotrichi*, and *Erysipelotrichales* (all belong to Firmicutes) (Figure 5C). We then compared the relative abundances of *Lactobacillus*, *AF12*, *Allobaculum* and Proteobacteria among the six groups and found a significant reduction in *Lactobacillus* and enrichment in *AF12* in the neonatal *S. pneumoniae*-infected group during infancy (Figure 5D,E), and a marked abridgment of *Allobaculum* and enrichment of *AF12* and Proteobacteria in adulthood (Figures 5D–G). Those results demonstrated that neonatal *S. pneumoniae* infection induced a significant transformation in gut microbial composition—primarily in infancy—and that the gut microbiome partially recovered by adulthood.

### Gut microbial dysbiosis-related pathway prediction

To determine the importance of neonatal *S. pneumoniae* infection-induced microbial dysbiosis in infancy, we exploited Phylogenetic Investigation of Communities by Reconstruction of Unobserved States (PICRUSt2) to conduct functional predictions for gut microbiota. When we compared the genetic composition of the gut microbiota of the mock-infected control and neonatal *S. pneumoniae*-infected mice in infancy, our results indicated that the metabolism- and degradation-related pathways were visibly enriched (Supplementary Figure 5). We also ascertained that the beta-alanine metabolism- and toluene-degradation pathways were significantly affected by neonatal *S. pneumoniae* infection in infancy relative to the control group.

## Discussion

Early life is a critical developmental window for gut microbial development, the disruption of which during maturation substantially perturbs immune- and metabolic- system evolution (Cunningham et al., 2014; Houghteling and Walker, 2015). Although exposure to antibiotics has been widely accepted to be a risk factor in microbial dysbiosis and asthma genesis (Tanaka et al., 2009; Ding et al., 2019), the effect of bacterial infection as an indication of antibiotic use has been neglected. Recently, the cross-talk between respiratory system and commensal organism, and it’s role in pulmonary disease are hot off the press. It is thought that gut microbial dysbiosis could produce adverse effect to innate lung immunity and increasing lung inflammation (Samuelson et al., 2015), contributing to asthma development (Depner et al., 2020). On the other side, infant respiratory infection (Harding et al., 2020) and neonatal hyperoxia exposure (Althouse et al., 2019) were reported associated with gut microbial community alteration. Those indicated that gut-lung axis is essential for homeostasis and host immune system training, may associated with chronic disease development and long-term health. We therefore adopted a conventional mouse model of neonatal *S. pneumoniae* infection that was reported to induce airway hyperresponsiveness (AHR) (Peng et al., 2019; Li et al., 2021) and experimental asthma (Yang et al., 2015; Tian et al., 2020), so as to explore the effect of neonatal bacterial infection on the gut microbiota. Although little influence was found on the total richness or uniformity of gut microbiota after neonatal *S. pneumoniae* infection, we observed a clear difference in gut microbial community structure and composition. This study presents a new perspective on the impact of early-life bacterial respiratory infections on gut microbial development and may lead to a better elucidation of the gut microbiome’s effect on lifelong health.

Gut microbial colonization begins at the moment of birth and fluctuates with age (Del Chierico et al., 2015). In this study we thus evaluated the dynamic changes of the gut microbiota in control mice and mice neonatally infected with *S. pneumoniae* at different developmental stages. Without detectable *S. pneumoniae* in gut (data not shown), we ascertained that alpha diversity increased with age during development and reached stability in adulthood, as revealed by the increased Shannon indices. The relative abundance of Bacteroidetes increased with age, and Firmicutes and Bacteroidetes ranked as the top two phyla. These results thus confirmed the rules governing gut microbial development, and were consistent with other studies in mice and humans (Tasnim et al., 2021).

After adjusting for age, we determined that neonatal *S. pneumoniae* infection disrupted gut microbial structure and composition using PCoA and LEFSe analyses, and found that this disruption occurred gradually but endured. One week after infection, *Lactobacillus* only showed a decreased trend in the breastfeeding period, while this reduction was amplified in infancy and then recovered until adulthood. Indeed, the reduction in *Lactobacillus* was an indication of gut microbial dysbiosis. *Lactobacillus* belongs to the larger lactic-acid bacterial group, which potentially influences the behavior of immune cells, and acts as an immune adjuvant and cellular forge that synthesizes and secretes bioactive molecules (Cho et al., 2020). In the presence of *L. plantarum* (a species of *Lactobacillus)*, macrophages produced lesser amounts of TNF-α, IL-1β, and IL-17; and peripheral blood mononuclear cells (PBMCs) produced more IL-10 (Ferreira Dos Santos et al., 2016). Other than their anti-inflammatory activities, *Lactobacillus* bacilli also influence immune-cell maturation and differentiation. *L. rhamnosus* and *L. plantarum* (two species of *Lactobacillu*s) increased the population of Th1 cells and Tregs in systemic lupus erythematosus-mouse models to reverse the disease profiles (Ivanovic et al., 2015). However, in chronic inflammatory disease, *Lactobacillus* bacilli tend to reduce Th activity, including Th17 cells (Chen et al., 2016). In addition, a positive role for early-life *Lactobacillus* abundance in asthma development has been confirmed in a large number of cohort studies of children (Björkander et al., 2020). These investigators determined that the absence of *Lactobacillus* species at six months of age induced elevated levels of chemokines that preceded allergic disease, and was associated with lower IFN-γ concentrations, higher fractional exhaled nitric oxide, and a higher incidence of allergic diseases. In our previous study we demonstrated that neonatal *S. pneumoniae* infection attenuated IL-10 and elevated IL-17 cytokines in bronchoalveolar lavage fluid (BALF), and reduced Th1 cells but enhanced pulmonary Th17 populations in adulthood, and that these alterations were confirmed to be contributors to the development of allergic airway disease (AAD) in adulthood (Yang et al., 2015; Tian et al., 2020). This may indicate that neonatal *S. pneumoniae* infection-induced *Lactobacillus* reduction in infancy comprises an essential component of gut microbial dysbiosis, which may in turn contribute to AAD development in adulthood.

Dysbiosis in infancy, however, does not impede the influence of *S. pneumoniae* infection on the gut microbiota, as we also found significant alterations in the gut microbial community during adulthood; this included increased Proteobacteria and decreased *Allobaculum* in neonatal *S. pneumoniae*-infected mice. Proteobacteria comprises a type of microbial dysbiosis signature that is reported to explain multiple pathogenic mechanisms (Lin et al., 2020). The phylum Proteobacteria is represented by potentially pathogenic bacteria—including *Haemophilus, Moraxella, and Neisseria* genera—that delineate toxin- and biofilm-producing opportunistic pathogens (Huang et al., 2015; Caverly et al., 2019). A higher abundance of Proteobacteria was uncovered in severely asthmatic patients, and this expansion was correlated with a Th17-cell epithelial-gene signature and was further correlated with asthma severity (Huang et al., 2015). Similar characteristics were unveiled in a mouse model, as *Moraxella*-infected mice showed augmented levels of IL-6, IL-1β, IL-17, and tumor necrosis factor α (TNF-α) in lungs, and neutralization of IL-17 and TNF-α was sufficient to accelerate airway inflammation and reduce the risk of AAD (Alnahas et al., 2017). Our previous work suggested that IL-17 acted as an essential cytokine in AAD development, indicating that proteobacterial expansion in gut microbiota could facilitate airway inflammation and AAD development. In contrast, *Allobaculum* is a genus of mouse commensal bacteria similar to *Faecalibacterium*, manifests the ability to assimilate glucose and produce short-chain fatty acids (SCFAs) (Greetham et al., 2004; Balakrishnan et al., 2021), such as butyrate, to exhibit immunoregulatory ability. *Allobaculum* was recently reported benefit for arthritis, which maintains immune and gut homeostasis through butyrate production augment (Balakrishnan et al., 2021). Functional prediction also consistently showed that metabolic and degradation-related pathways were markedly enriched in our model. Additionally, beta-alanine and toluene were both reported related with airway obstruction or asthma development (Wang et al., 2021), which might be an indication for our further mechanism exploration. Thus, the changes we noted in mice neonatally infected with *S. pneumoniae* suggest long-lasting disruption of gut microbiota by bacterial respiratory infection, and may correlate with health conditions in later life.

The findings from this study suggested that bacterial respiratory infection in mouse neonates produced a profound effect on the gut microbiota, and that although this effect occurred gradually, it continued into adulthood. In the context of respiratory disease, most of the studies focused on the effect of gut microbial dysbiosis to lung immunity, Alterations of the gut microbial community may be essential for the immune and metabolic systems, and this might explain diseases that occur in later life. On the other side, with the hypothesis of gut-lung axis, investigators come to realize the influence of virus respiratory infection on gut microbiota (Deriu et al., 2016; Bartley et al., 2017). Groves et al. (2020) reported airway RSV infection induced CD8 + cytokine production, which may inhibit appetite of mice and contribute to gut microbial alteration. Consistent with that, we previously found loss of appetite and weight in our neonatal *S.pneumoniae* infection model, indicating the possible mechanism in our investigation.

There were several limitations to this study. First, although 16S rRNA high-throughput gene sequencing identified the majority of bacteria, genome sequencing and metabonomics are still needed to allow further progress in understanding the underlying mechanisms of action. We also found a significant gut microbiota dysbiosis after neonatal *S. pneumoniae* infection, and further microbial community restoration is therefore required to fully investigate the relationships between microbial disruption and AAD development. Finally, due to medical ethics, this hypothesis could only be applied to a mouse model, even though mice have been thought to constitute a well-recognized model for human trials. As there are, of course, species differences in the gut microbiota of humans and mice, additional studies are required to better clarify the effects of neonatal bacterial respiratory infection on microbiome development in humans.

## Conclusion

In summary, our results first highlighted the substantial influence of neonatal bacterial respiratory infection in neonates on the gut microbiota. Neonatal *S. pneumoniae* infection not only influenced gut microbial diversity and community, it also promoted a long-lasting modification of microbial community composition, and this was particularly true for the reduction in immune/metabolic regulatory flora such as *Lactobacillus* and *Allobaculum*. These findings will better elucidate bacterial respiratory infection in early life on gut microbiota development, and clarify its effects on lifelong health.

## Data availability statement

The data presented in this study are deposited in the NCBI repository, accession number: PRJNA858952 (https://submit.ncbi.nlm.nih.gov/subs/sra/SUB11796996/overview).

## Ethics statement

The animal study was reviewed and approved by the Institutional Animal Care and Research Advisory Committee of the Chongqing Medical University.

## Author contributions

ZL conceived and designed the experiments. YL drafted the manuscript. YL, XX, and ZL revised the manuscript. YL, YW, DJ, and ZG performed the experiments. YL, GZ, XT, and XX analyzed the data. All authors contributed to the article and approved the submitted version.
