# Supplementary material for: Neonatal Streptococcus pneumoniae infection induces long-lasting dysbiosis of the gut microbiota in a mouse model
Source: Front Microbiol. 2022 Aug 18;13:961684. doi: 10.3389/fmicb.2022.961684 (PMC9433971; doi:10.3389/fmicb.2022.961684)
Supplement: Supplementary file 1 [file Data_Sheet_1.docx]

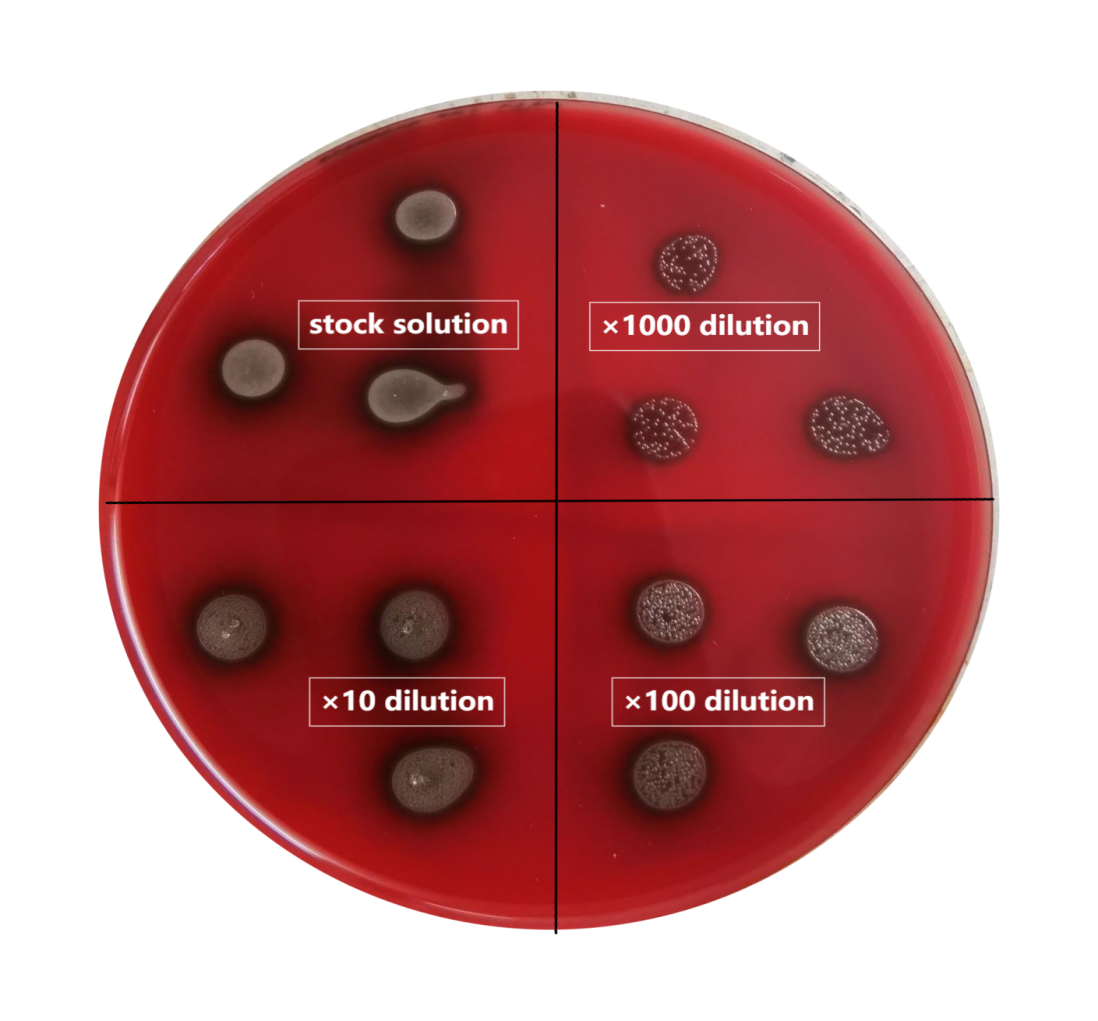


**Figure S1.** Bacterial number determination in lung tissue homogenate.

**Figure S2. Gut microbial community structure changed with age, and varied with neonatal S. pneumoniae pneumonia in the same stage.** The differentiation of microbiota structure between mock-infected control- and neonatal *S. pneumoniae* pneumonia (*S*. pp)-treated mice in the breastfeeding period (1 wpi), infancy (2 wpi), and adulthood (7 wpi) is compared by PERMANOVA based on Bray-curtis metrics.


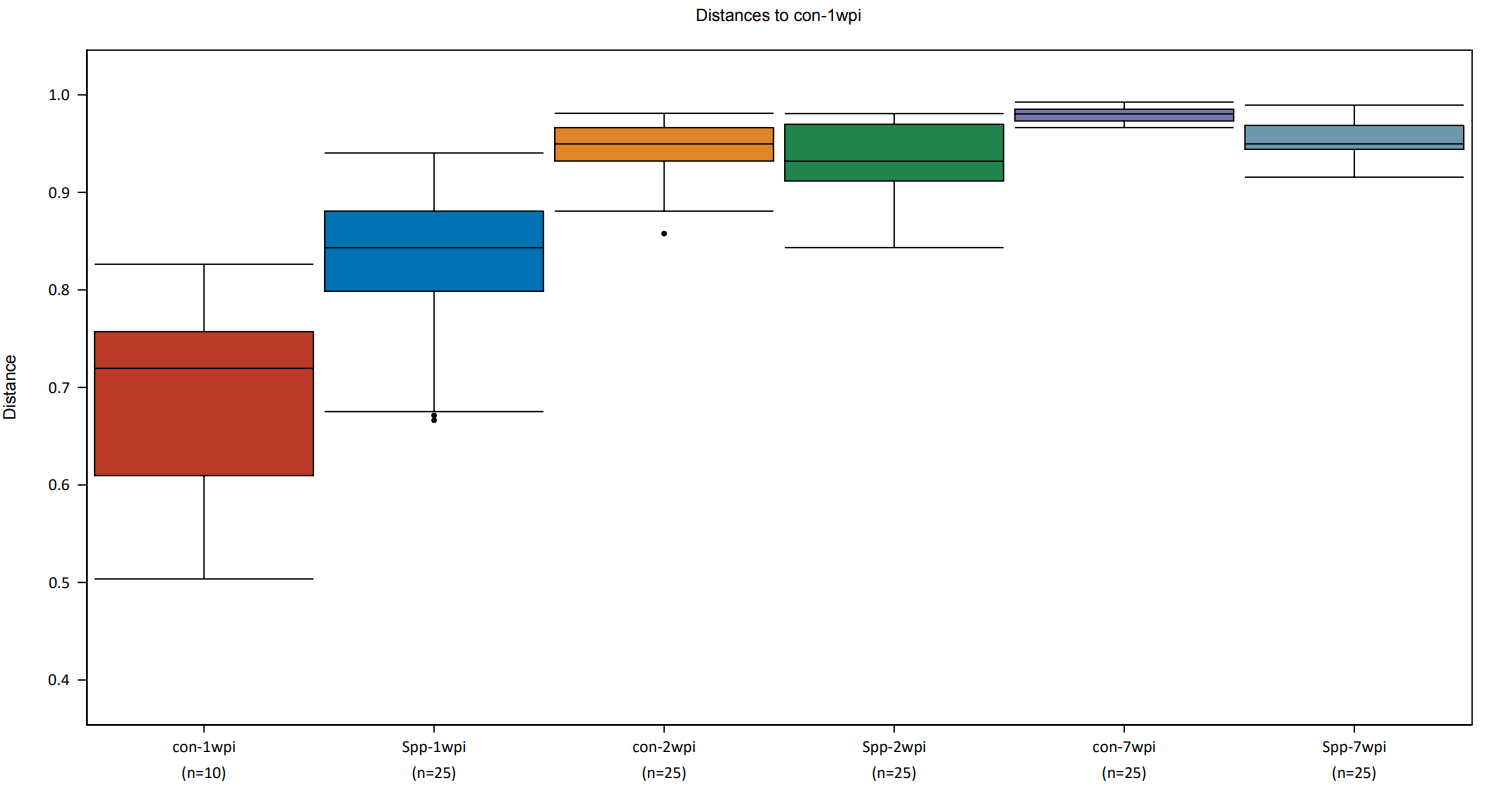


**Table S1.** PERMANOVA test among groups

| Group 1 | Group 2 | Sample size | Permutations | Pseudo F | P value | Q value |
| --- | --- | --- | --- | --- | --- | --- |
| all |  | 30 | 999 | 4.45 | 0.001* | - |
| Con 1wpi | S.pp 1wpi | 10 | 999 | 3.55 | 0.009* | 0.012* |
| Con 1wpi | Con 2wpi | 10 | 999 | 3.78 | 0.011* | 0.012* |
| Con 1wpi | S.pp 2wpi | 10 | 999 | 7.90 | 0.01* | 0.012* |
| Con 1wpi | Con 7wpi | 10 | 999 | 5.41 | 0.005* | 0.012* |
| Con 1wpi | S.pp 7wpi | 10 | 999 | 4.27 | 0.006* | 0.012* |
| S.pp 1wpi | Con 2wpi | 10 | 999 | 4.28 | 0.009* | 0.012* |
| S.pp 1wpi | S.pp 2wpi | 10 | 999 | 9.41 | 0.007* | 0.012* |
| S.pp 1wpi | Con 7wpi | 10 | 999 | 5.70 | 0.009* | 0.012* |
| S.pp 1wpi | S.pp 7wpi | 10 | 999 | 4.37 | 0.01* | 0.012* |
| Con 2wpi | S.pp 2wpi | 10 | 999 | 3.05 | 0.011* | 0.012* |
| Con 2wpi | Con 7wpi | 10 | 999 | 2.59 | 0.012* | 0.012* |
| Con 2wpi | S.pp 7wpi | 10 | 999 | 2.01 | 0.004* | 0.012* |
| S.pp 2wpi | Con 7wpi | 10 | 999 | 6.02 | 0.009* | 0.012* |
| S.pp 2wpi | S.pp 7wpi | 10 | 999 | 5.74 | 0.002* | 0.012* |
| Con 7wpi | S.pp 7wpi | 10 | 999 | 2.55 | 0.012* | 0.012* |

* significant difference between groups


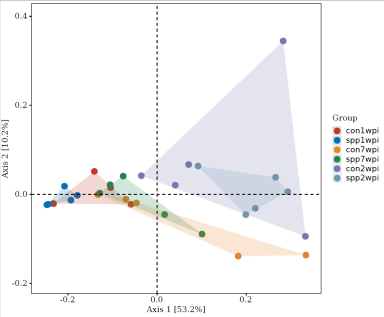

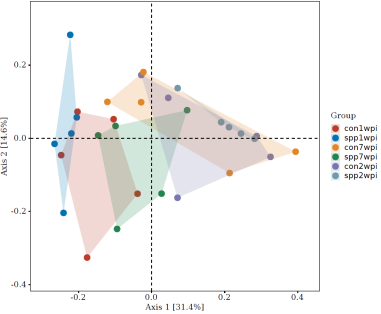

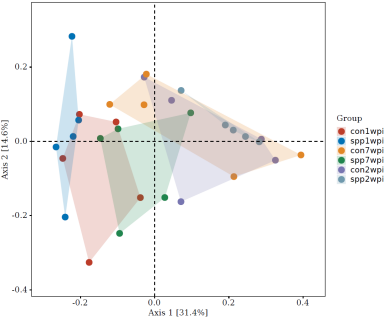

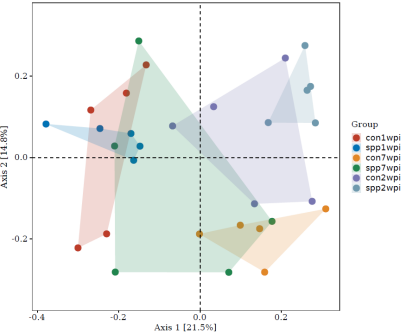

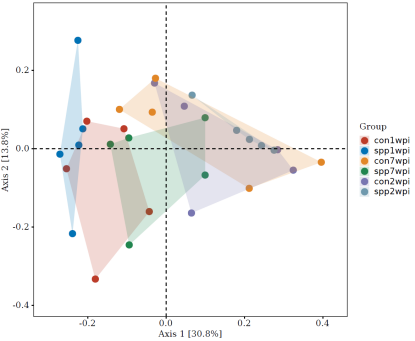

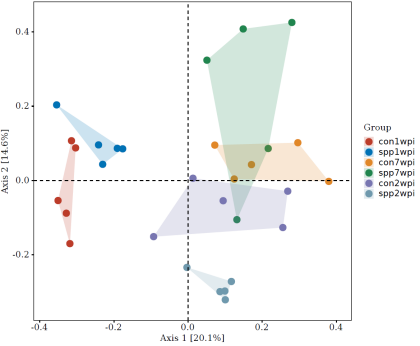


(a)

(b)

(c)

(d)

(e)

(f)

**Figure S3. Beta-diversity analysis gut microbiota at different levels.** Beta-diversity analysis of relative abundances of the Phylum (a), Class (b), Order (c), Family (d), Genus (e), Species (f) using principal coordinate analysis (PCoA) with Bray-Curtis dissimilarity index.


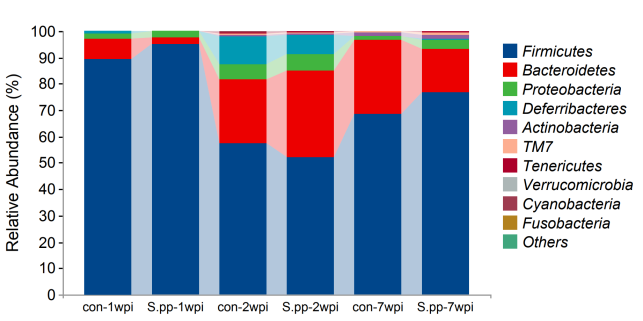


**Figure S4. Effects of neonatal *S. pneumoniae* infection on gut microbial composition at the phylum level.** Relative abundances at the Phylum level of the mock-infected control and neonatal *S. pneumoniae* pneumonia (*S*. pp) groups in the breastfeeding period (1 wpi), infancy (2 wpi), and adulthood (7 wpi).


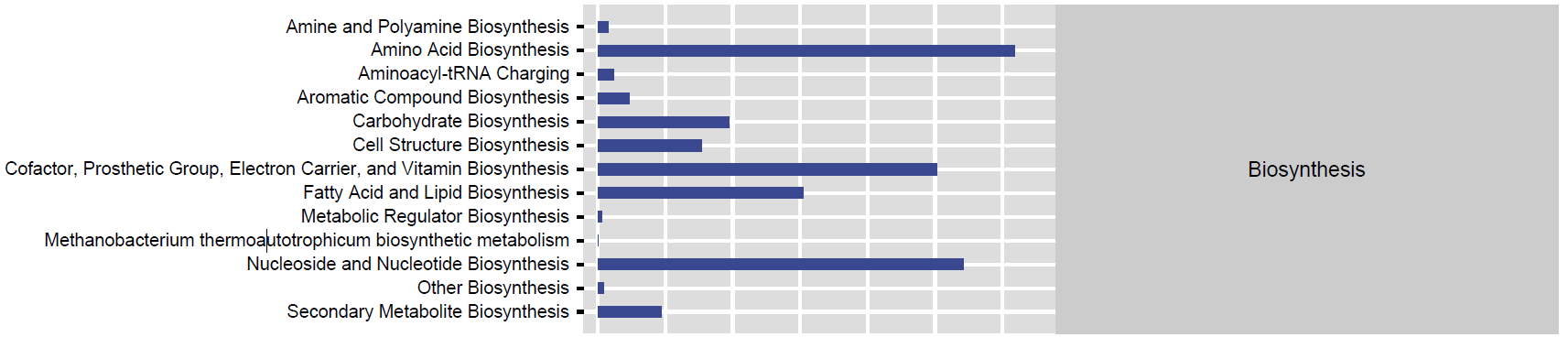

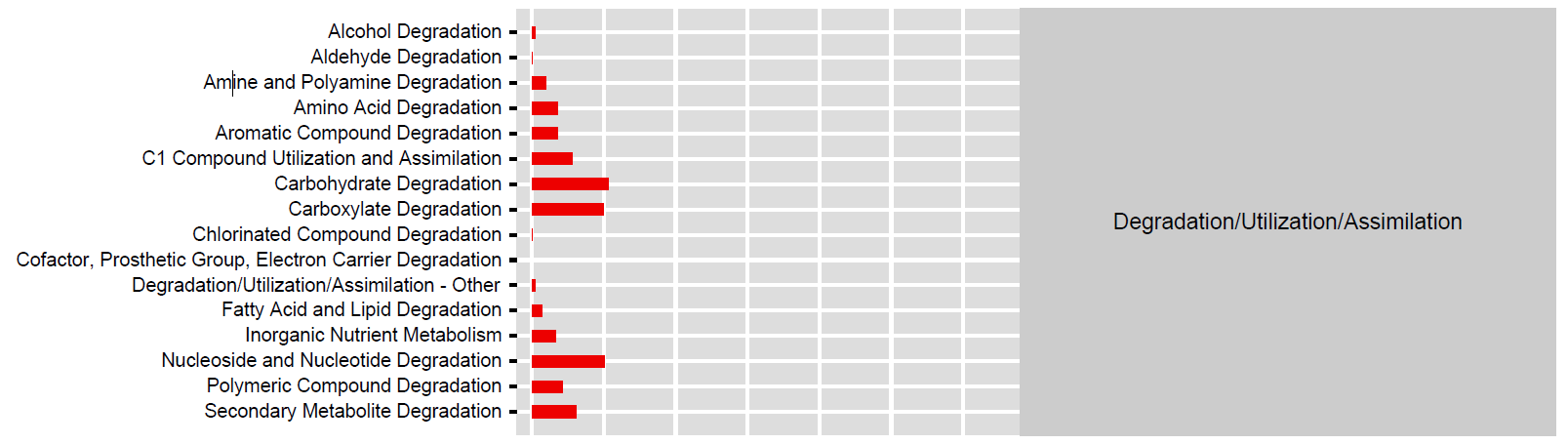

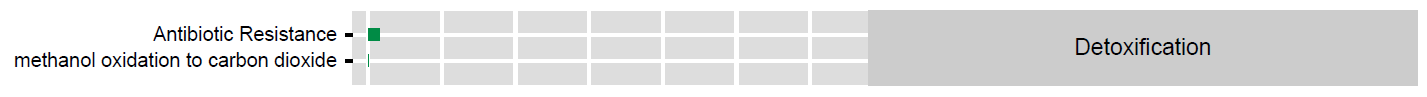

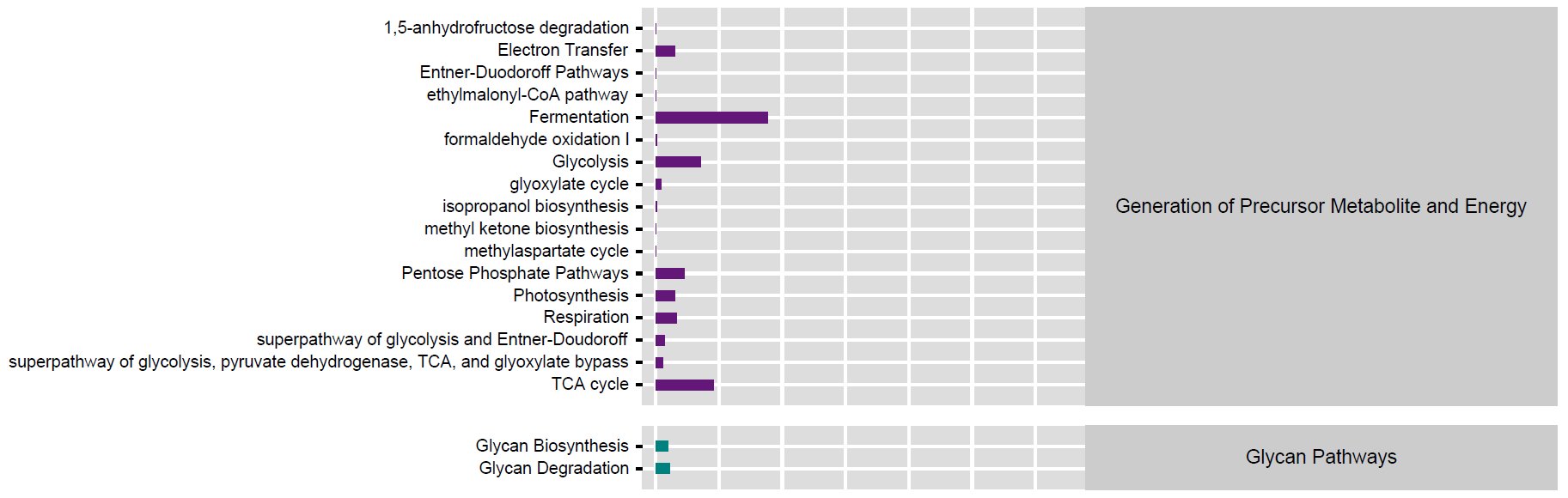

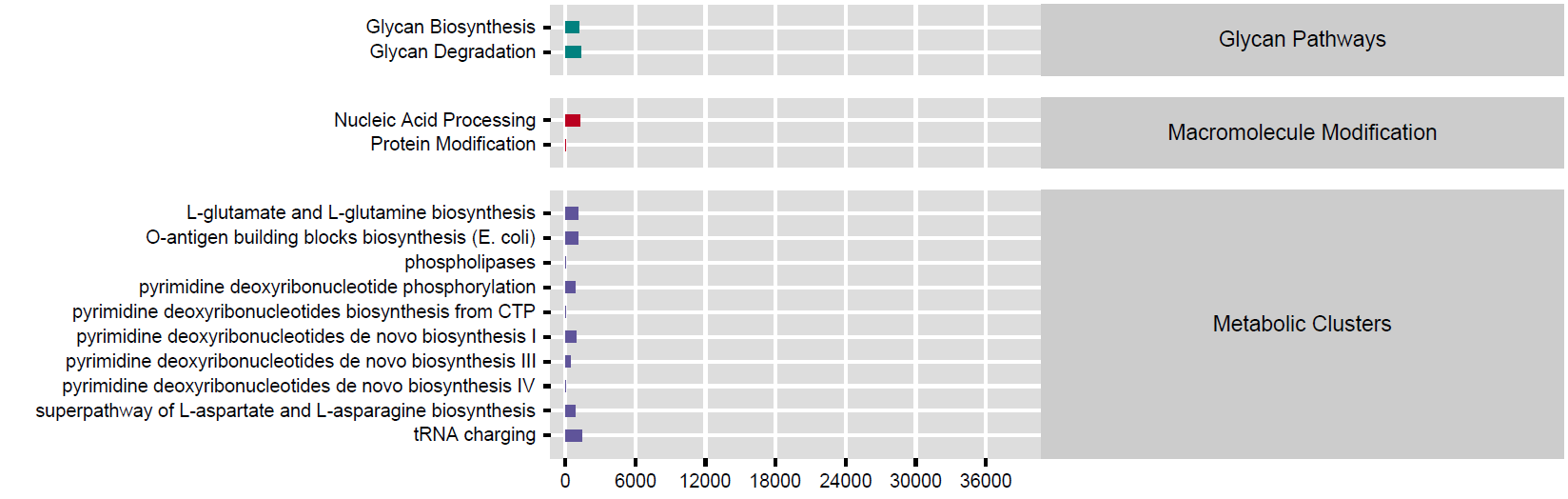


**Figure S5.** **Functional classification of control and neonatal *S. pneumoniae* infection mice.** Functional classification of the predicted metagenome content of the microbiota of the infant mock-infected control and neonatal *S. pneumoniae* infection (S. pp) groups using Metacyc.
